# On sustainability and low value care

**DOI:** 10.1080/02813432.2024.2321525

**Published:** 2024-02-28

**Authors:** Hálfdán Pétursson, Margrét Ólafía Tómasdóttir

**Affiliations:** a Department of Family Medicine, Faculty of Medicine, University of Iceland, Reykjavik, Iceland; b Research and Development Primary Health Care Gothenburg and Södra Bohuslän, Region Västra Götaland, Sweden; c Development Centre for Primary Healthcare in Iceland, Reykjavik, Iceland

The term *sustainability* has in recent years rightfully received increasing attention in relation to environmental and planetary sustainability. The field of medicine has not been left untouched by this trend, although the great threat of global warming calls for a more significant change in mindset if meaningful progress is to be made [1]. Planetary health is, however, only one aspect of sustainability of the healthcare system. Increased longevity and changing demographics with a growing population of the elderly and a rising prevalence of frailty and multimorbidity alongside an expanding arsenal of expensive treatment options cannot forever be met by the proceeding trend of enlarging healthcare budgets. Continuing expansion of the healthcare system with rising expenditure is not a sustainable development and there is an obvious necessity of prioritising our resources better. Furthermore, there is a worldwide shortage of healthcare personnel which puts further restrictions to the capabilities of healthcare systems to meet these increasing demands.

Minimising low value care is a mandatory task when considering possible ways to secure the sustainability of our healthcare system. Practices and procedures that offer no or very limited clinical effect, have a poor risk-benefit profile or are not adequately supported by evidence are common and consume a considerable proportion of the healthcare budget [2]. Low value care has been estimated to account for one-fifth or even more of the resources spent on healthcare [3]. There is a myriad of practices that constitute low value care. Examples can, for instance, be found in prescribing patterns of antibiotics [4], SSRIs and ADHD medication, as well as in the abundance of laboratory tests [5,6] and medical imaging ordered. Low value care is not only wasting our valuable resources but causing harm to patients as well; directly through mechanisms such as adverse effect of treatments and diagnostic procedures, and indirectly through opportunity costs, leading to longer waiting lists for important interventions and care, prioritising people in less need for service above those in greater need, in accordance with the inverse care law [7].

There is a common misunderstanding among policy makers, the public, and even colleagues, that MORE healthcare automatically leads to better health – disregarding the law of diminishing returns [8]. The notion that easier access to care is inherently desirable may obscure what is being down-prioritised. For instance, increasing access to out of hours services and digital contacts might not improve access for those in greatest need of care and may affect continuity of care negatively by displacement of personnel from long-term follow-up to walk-in services. The notion that more information – offered by laboratory tests, medical imaging and screening procedures – and earlier diagnosis are automatically beneficial disregards the threats of overdiagnosis and false positive results. These notions are among the drivers of overdiagnosis and low value care.

Quite often there seems to be an insufficient understanding or recognition of the fact that resources allocated to practices of limited value have to be withheld from procedures of greater value. Evidence (or strong belief) of cost-effectiveness does not make an intervention exempt from being down-prioritised in favour of practices of more value. Guidelines recommending increasingly aggressive treatment goals and lower thresholds for initiating treatment – such as guidelines on cardiovascular risk prevention – without adequate consequence analyses, are clear examples of this. This is also the case when widespread screening for risk factors is suggested, or even risk factors for risk factors. Interventions at lower levels of risk are bound to offer lower value in return, treating more people with lower chance of benefit but stable risk of adverse effects. However, the value of a certain practice may be deemed differently by different individuals. The value, at least to some extent, lies in the eye of the beholder.

Preventing overdiagnosis and overtreatment, as well as prioritising those whose needs for healthcare are the greatest, are among the core values of general practice. The list of core values, as presented by the Nordic Federation of General Practice and WONCA Europe, is thus in strong and explicit opposition to low value care [9]. There is a compelling need for prioritising sustainability in policy and practice, as well as in research. Market-driven solutions have become more prevalent, such as direct to consumer access to diagnostic tests and imaging, direct online contact with a physician on video or chat for minor problems. The chase for quick solutions has, for example, been crystallised in the explosive sales of GLP-1 analogues. Are we truly spending our time and resources in the best possible way to benefit the health of our population? Or is that perhaps no longer the overarching aim of healthcare?

Various initiatives against low value care have been initiated in the Nordic countries. Official national initiatives based on Choosing Wisely are running in Norway and Denmark and the process has been started in Sweden. Great progress has been made in recent decades towards decreasing antibiotic resistance by more appropriate prescribing patterns. But other prescribing statistics, such as for opioids and proton pump inhibitors, show that more such initiatives are needed [10]. GPs are in an excellent position to lead the way towards more sustainable practice. Holding the core values of general practice high is of utmost importance on this voyage. Analysing one’s own clinical practice can be a useful starting point for the individual GP. But the views and voices of primary care need to have greater impact on policy decisions. We need to engage in a dialogue on multiple levels to promote sustainability instead of a zero-vision; a dialogue within as well as between professions, with policy makers, and with the society. Researchers in primary care should take the moral responsibility to strive for sustainability through their research. Studies on themes such as de-implementation [11], continuity of care [12] and horizontal prioritisation [13] are more needed than risk factor research that tends to define an ever increasing proportion of the population at risk and in need of care.

A sustainable and fair healthcare system demands responsible allocation of resources for the greatest possible benefit of public health while prioritising those whose needs are the greatest. A sincere and continuous effort to identify and reduce extent of low value care is a mandatory component of the quest for sustainability.
